# The critically ill older patient with sepsis: a narrative review

**DOI:** 10.1186/s13613-023-01233-7

**Published:** 2024-01-10

**Authors:** Mercedes Ibarz, Lenneke E. M. Haas, Adrián Ceccato, Antonio Artigas

**Affiliations:** 1 Department of Intensive Care Medicine, Hospital Universitari Sagrat Cor, Quirón Salud, Viladomat 288, 08029 Barcelona, Spain; 2 Department of Intensive Care Medicine, Diakonessenhuis Utrecht, Utrecht, the Netherlands; 3 Department of Intensive Care Medicine, CIBER Enfermedades Respiratorias, Corporación Sanitaria Universitaria Parc Tauli, Autonomous University of Barcelona, Sabadell, Spain; 4 Institut d’investigació i innovació Parc Tauli (I3PT-CERCA), Sabadell, Spain

**Keywords:** Sepsis, Old, Very old, Intensive care, Infection outcomes

## Abstract

Sepsis is a significant public health concern, particularly affecting individuals above 70 years in developed countries. This is a crucial fact due to the increasing aging population, their heightened vulnerability to sepsis, and the associated high mortality rates. However, the morbidity and long-term outcomes are even more notable. While many patients respond well to timely and appropriate interventions, it is imperative to enhance efforts in identifying, documenting, preventing, and treating sepsis. Managing sepsis in older patients poses greater challenges and necessitates a comprehensive understanding of predisposing factors and a heightened suspicion for diagnosing infections and assessing the risk of sudden deterioration into sepsis. Despite age often being considered an independent risk factor for mortality and morbidity, recent research emphasizes the pivotal roles of frailty, disease severity, and comorbid conditions in influencing health outcomes. In addition, it is important to inquire about the patient's preferences and establish a personalized treatment plan that considers their potential for recovery with quality of life and functional outcomes. This review provides a summary of the most crucial aspects to consider when dealing with an old critically ill patient with sepsis.

## Background

Despite advances in modern medicine, sepsis remains a major cause of morbidity and mortality. Sepsis accounts for 20% of global deaths [1], and survivors often endure long-term physical, psychological, and cognitive impairments.

Reporting sepsis epidemiology accurately is challenging due to evolving definitions, variations in reporting, demographic disparities, and discrepancies in healthcare resources [2, 3]. Estimates of sepsis cases range widely, from 19 to 48.9 million yearly [3, 4].

**Fig. 1:**
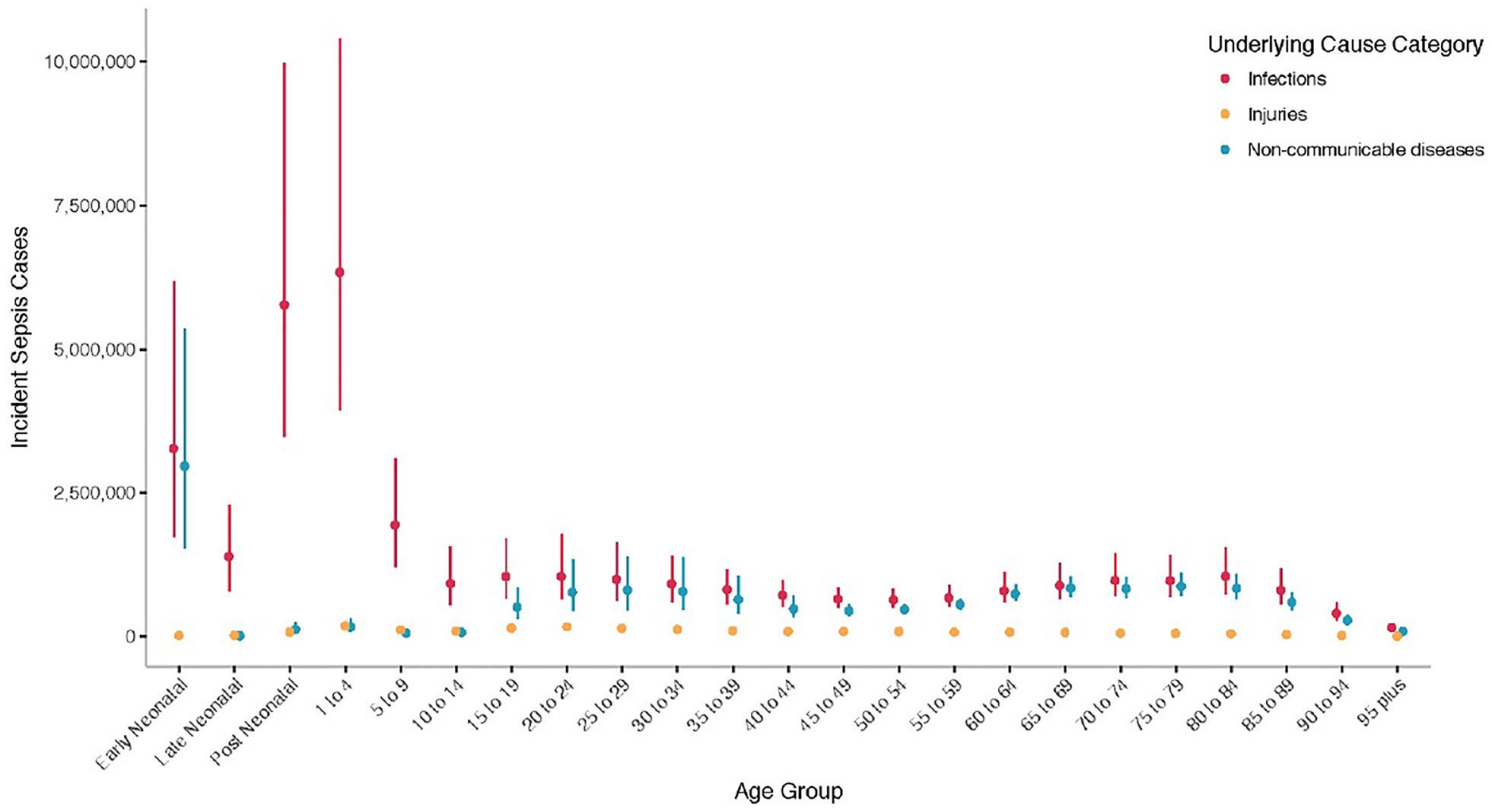
Incident sepsis cases by age group and underlying cause category, both sexes, all locations, 2017. Bars represent 95% uncertainty intervals. Reproduced from (1). Published under the CC BY 4.0 license

**Fig. 2:**
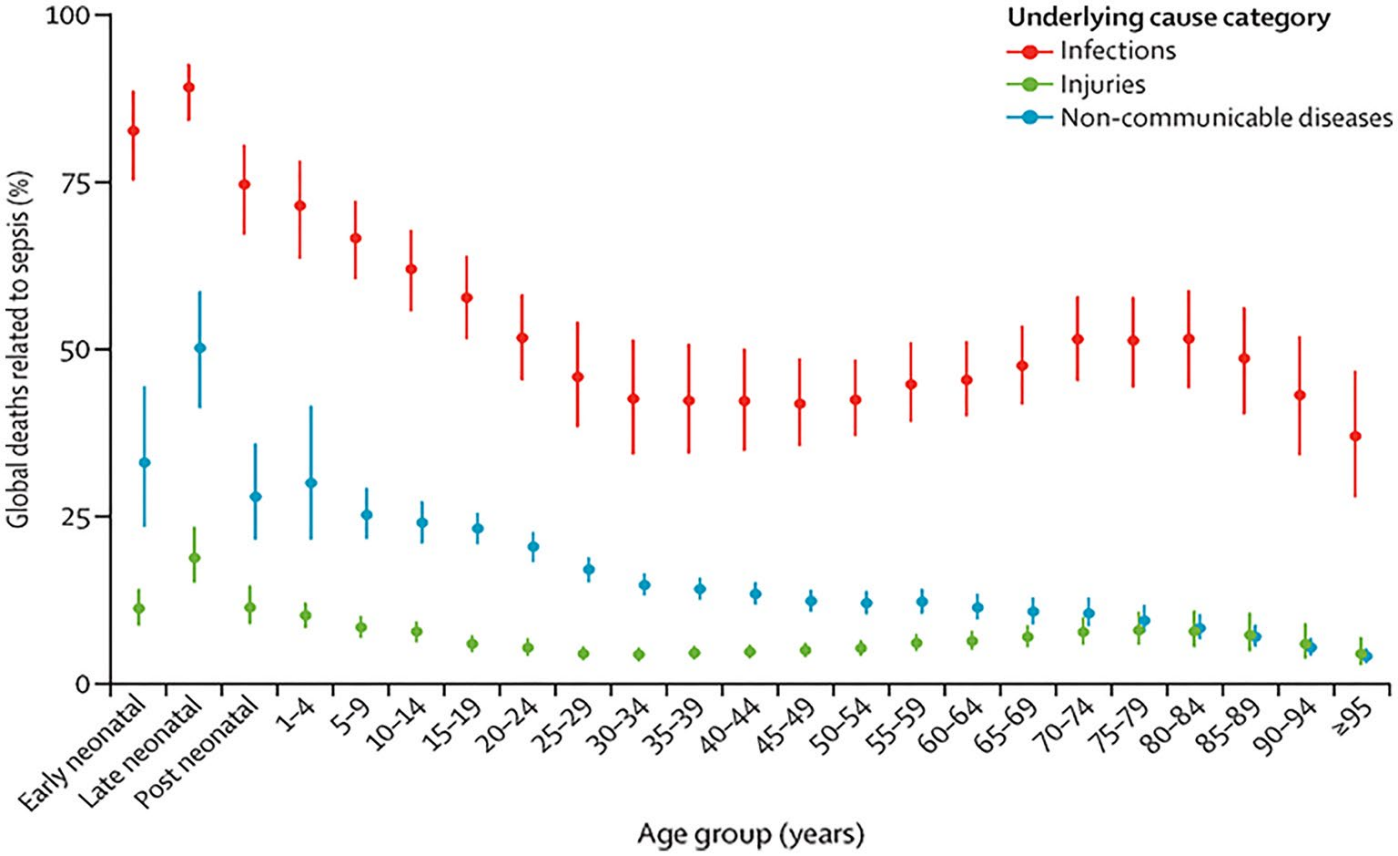
Percentage of all sepsis-related deaths in each underlying cause category, by age group and for both sexes, in 2017. Bars represent 95% uncertainty intervals. Reproduced from (1). Published under the CC BY 4.0 license

According to the Centers for Disease Control and Prevention, at least 1.7 million adults in the U.S. develop sepsis each year, resulting in nearly 270,000 deaths. Global sepsis data analysis [1] indicates a significant rise in sepsis cases, reaching 11 million deaths and 48.9 million incident cases in 2017 (Figs. 1, 2). While age-standardized sepsis incidence dropped by 37.0% and mortality by 52.8% between 1990 and 2017, substantial regional differences persist. The study highlights a decrease in global sepsis burden but emphasizes the urgent need for intervention, particularly in areas with the lowest Socio-Demographic Index.

In 2021–22, England and Wales reported over 100,000 emergency admissions with sepsis, with a mean patient age of 71 years [5]. In England, sepsis represented one-third of admissions to adult ICUs [6] and in China sepsis affected a fifth of patients admitted to the ICU [7].

Sepsis affects all age groups, but its incidence and mortality notably increase with advancing age, particularly in older adults who face elevated risks [8, 9]. In a Taiwanese nationwide study on sepsis, the incidence of sepsis in the oldest old (≥ 85 years) was 31-fold greater than the adult incidence (18–64 years) and threefold greater than the old (65–84 years) [10].

Due to an aging population, sepsis incidences are expected to rise. By 2050, about 16% of the global population will be aged 65 and above [11]. The most rapid increases in older populations are happening in developed countries, with a projected 140% rise in individuals aged 65 years and older by 2030, and those aged 85 years and above being the fastest-growing group [11–13].

Today, three key factors stand out: a global increase in sepsis cases [1, 14], significant healthcare challenges from sepsis-related mortality and morbidity [2, 15] and a notably rise in very old patients with sepsis due to the aging of population [16, 17]. Addressing these challenges requires standardized definitions, improved data collection, and better healthcare access.

In this review we will underscore the factors that contribute to the increased susceptibility to sepsis and higher mortality risk in older patients. The focus advocates for a comprehensive strategy in sepsis management, emphasizing a holistic approach and personalized care that considers individual factors, such as frailty, comorbidities, and patient values. We consider "older adults" as those surpassing 65 years with 'very old' individuals being those over 85 years.

### Risk factors for sepsis

**Fig. 3:**
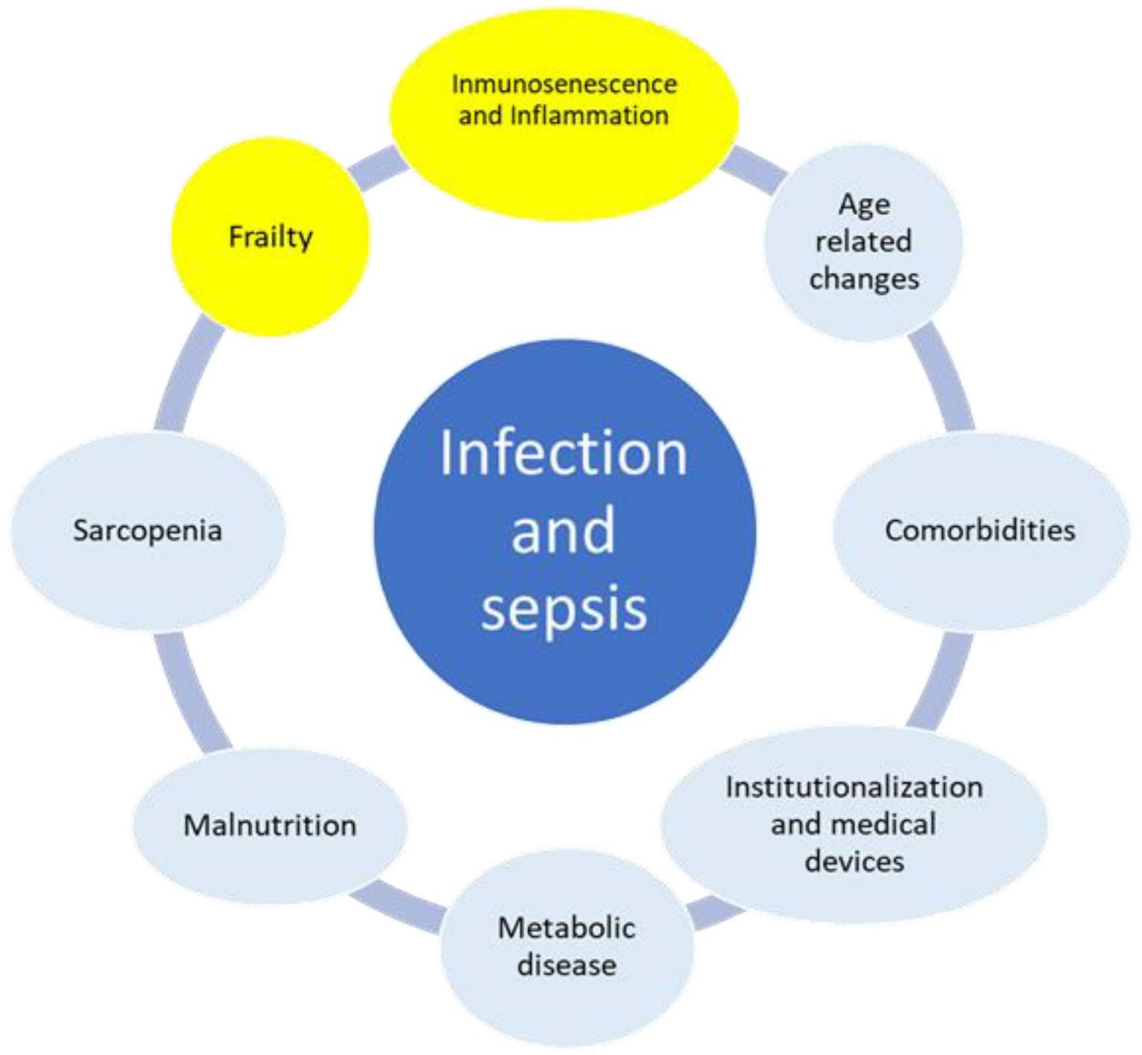
Risk factors for sepsis in older adults. Older adults face an elevated risk of sepsis due to several factors, including aging itself, comorbidities, and a weakened immunity. The interplay between their general health and sepsis severity significantly influences both short- and long-term outcomes, emphasizing the need for comprehensive assessment and personalized treatment strategies

Older individuals are particularly vulnerable to developing sepsis due to pre-existing comorbidities, compromised immune function, sarcopenia, diminished physiological reserves associated with aging, malnutrition, and polypharmacy (Fig. 3). In the subsequent discussion, we will focus on the key factors, with additional details available in a recent review [18].

*Immunosenescence and inflammaging* play a crucial role in making the older individuals more susceptible to sepsis [19, 20]. Immunosenescence involves a gradual decline in the immune system, especially T-cell function and inflammaging is characterized by persistent low-grade inflammation. Both processes are interconnected, forming a cycle that heightens susceptibility [19, 21–23]. The immune system’s interaction with other systems, such as the neural or endocrine system, links declining immune function to frailty, sarcopenia, and malnutrition [19, 20]. Reduced insulation and lower metabolism compromise the immune system, making older individuals more vulnerable to infections and illnesses.

Geriatric syndromes arising from impairments in multiple systems, result from a combination of age-related changes, underlying medical conditions, and environmental influences and significantly impact quality of life and increase susceptibility to infection.

*Frailty,* a clinically recognizable state of increased vulnerability resulting from aging-associated decline, becomes more prevalent with age, impacting 25% of those over 65 and over 50% of patients over 80. It affects approximately 40% of older ICU patients and significantly impacts mortality and morbidity [24–30]. Incorporating frailty assessment into risk stratification can identify a vulnerable population that may benefit from targeted interventions.

*Sarcopenia*, characterized by muscle decline, has a prevalence rate ranging from 11 to 50% in those aged 80 years and above [31]. Aging disrupts muscle balance, triggering mechanisms, such as anabolic resistance, reduced IGF-1 signalling, mitochondrial dysfunction, inflammation, and oxidative stress, leading to muscle loss. Anabolic resistance diminishes muscle responsiveness to stimuli, causing reduced protein synthesis and muscle wasting. Immobilization in hospitalized older individuals results in a daily muscle mass reduction (0.5%) and strength decline (0.3–4.2%), impacting functional status and quality of life [32]. Sepsis worsens sarcopenia by promoting inflammation, muscle wasting, and potential mitochondrial dysfunction [33, 34]. Sarcopenia is linked to various pathophysiological processes, increasing mortality risk, especially in critical illness [33].

*Malnutrition and dehydration* are widespread in older people, and obesity is an increasing problem [35]. Malnutrition, linked to reduced food intake, underlying health issues, and nutrient absorption problems, contributes to functional decline, sarcopenia, slow wound healing, and adverse outcomes, such as increased infection rates and prolonged hospital stays [36]. Prevalence rates vary but can exceed two-thirds in hospitalized patients [35]. Dehydration prevalence can rise to over one-third in more vulnerable individuals [35]. Preventive measures, ensuring adequate nutrition and hydration, are essential. In hospital settings interventions such as a protein-rich diet, nutritional supplements, sedation protocols with short-acting drugs and early mobilization can improve outcomes. Routine screenings for prompt identification of potential malnutrition risks in geriatrics patients are recommended [35].

*Cognitive impairment*, is associated with brain changes, including reduced grey and white matter volume, impaired blood flow, altered neurotransmitter activity, and a more permeable blood–brain barrier [37]. It involves memory, attention, and cognitive deterioration potentially progressing to dementia at a rate of 10–15% per year. Critical illness often induces psychological symptoms, sleep disturbances, delirium, and cognitive impairment, all associated with higher mortality rates [38]. Delirium independently increases mechanical ventilation duration, ICU and hospital stays, health care costs, long-term cognitive impairment, and mortality risk. Non-pharmacological measures for delirium prevention are recommended [39].

*The impact of comorbidities* on septic patients is substantial. Malignancies, diabetes mellitus, and dysfunctions in cardiac, renal, liver, or pulmonary systems contribute to poorer outcomes. Notably, 78% of septic patients have at least one comorbidity [40], and 60% exhibit three or more [41]. On average, patients aged 65 to 84 have 2.6 ± 2.2 comorbidities, while those aged 85 or over have 3.6 ± 2.3 [42].

Moreover, older individuals face other vulnerabilities, including altered vaginal flora in women due to reduced estrogen levels, urinary issues from prostatic hypertrophy in men, compromised skin integrity, diminished cough reflex, and swallowing difficulties, all contributing to increased infection susceptibility. The use of *medical instruments and institutionalization* further heightens sepsis risk particularly due to the prevalence of multidrug-resistant (MDR) pathogens in healthcare settings.

Finally, aging shows significant individual heterogeneity, with some maintaining resilience and an active lifestyle, while others face higher susceptibility to diseases and disabilities. Understanding resilience, the ability to withstand and recover from stressors, is crucial for addressing chronic diseases and promoting healthy aging [43]. Lifestyle interventions, such as personalized exercise, and nutrition may help older individuals better adapt to the biological changes associated with aging and potentially reduce their susceptibility to infections [32, 44].

### Diagnosis of sepsis

Sepsis is a life-threatening organ dysfunction caused by a dysregulated host response to infection [45]. Organ dysfunction is defined as an acute increase of two or more points in the Sequential Organ Failure Assessment (SOFA) score [46]. Septic shock, a severe form of sepsis with circulatory, cellular, and metabolic dysfunction, carries a higher risk of mortality compared to sepsis alone [45, 47].

**Fig. 4:**
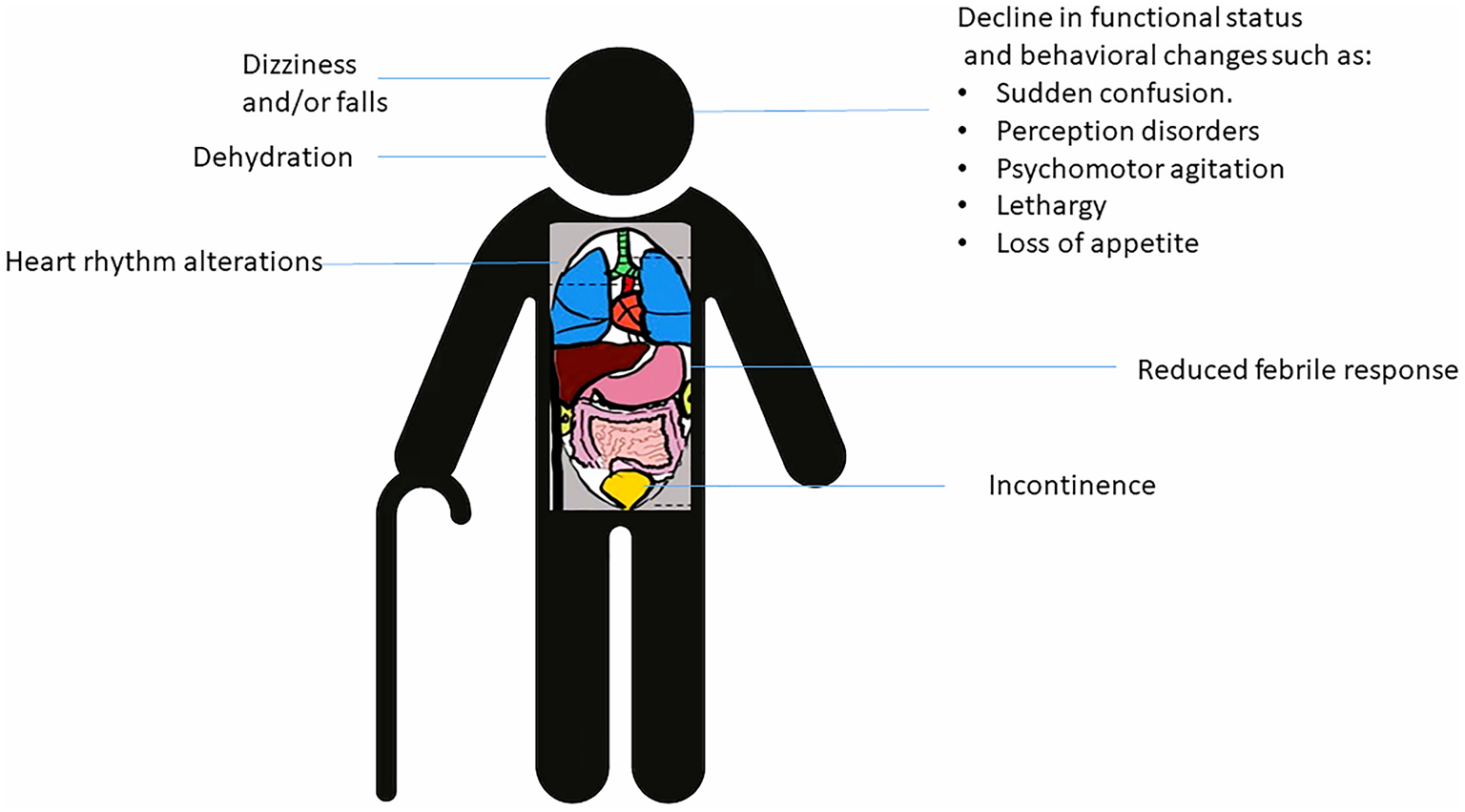
Clinical picture in older patients may be ambiguous

Diagnosing sepsis in older individuals can be challenging due to atypical presentations and subtle symptoms [48–50] (Fig. 4). Timely recognition is crucial for proper management and prevention of adverse outcomes. Therefore, a comprehensive evaluation, including a detailed history, thorough physical examination, and a heightened suspicion for infections is necessary. Biomarkers can provide fast and accurate early diagnosis compared to traditional microbiology tests, reducing the risk of negative results due to prior antibiotic treatment.

In older individuals, potentially life-threatening infections may manifest through various behavioural changes, including sudden confusion, perception disorders, psychomotor agitation, or lethargy. Physical symptoms such as loss of appetite, dehydration, dizziness, falls, and incontinence can serve as sole indicators. Notably, fever, a common sign of infection, is absent in 30–50% of older adults, who may exhibit a reduced febrile response to infections, such as bacteraemia, pneumonia, endocarditis, and meningitis [51–53]. The conventional definition of fever may not apply due to the lower baseline body temperature in older adults, influenced by diminished cytokine production, reduced hypothalamic receptor sensitivity, and impaired adaptation of peripheral thermoregulation [51, 54]. The use of medications, such as non-steroidal anti-inflammatory drugs, corticosteroids, beta-receptor blockers, antihistamines, and ranitidine, further dampens the inflammatory response. Therefore, assessing temperature changes from their baseline proves more useful than relying on absolute values.

Regarding organ dysfunction related to sepsis, the SOFA score serves as a tool to assess organ failure and functions both diagnostically and prognostically [45]. It is crucial to consider the interplay between pre-existing comorbidities and acute organ dysfunction when evaluating organ failure.

Biomarkers contribute to antibiotic stewardship by minimizing unnecessary prescriptions. However, their performance may differ in older patients due to comorbidities and chronic inflammation. Clinical judgment and comprehensive assessment are necessary when using biomarkers in this population [55].

*Lactate measurements* indicate tissue hypoperfusion and sepsis severity. Levels of 2 mmol/L and higher predict mortality regardless of age, but factors such as dehydration and anaemia, common in older individuals, can also increase lactic acid levels [56, 57]. Comorbidities, such as heart, liver, renal or respiratory dysfunction, may contribute to increased lactate levels due to factors, such as reduced cardiac output, impaired liver function, compromised kidney clearance, and inadequate tissue oxygenation elevating the risk of developing type B lactic acidosis or hinder lactate clearance.

*C-reactive protein* (CRP) has low specificity in this population. There is growing evidence suggesting that CRP is not only an inflammatory biomarker but is also associated with age-related conditions, such as cardiovascular disease, hypertension, diabetes mellitus, and kidney disease [58].

*Serum procalcitonin* (PCT) is a valuable biomarker for bacterial infections and sepsis prognosis [59–62]. It can be applied in older patients using similar cutoff values as in younger patients [63] demonstrating comparable performance and higher diagnostic accuracy than other markers [55]. Serial PCT measurements can guide antibiotic therapy duration, reducing exposure without compromising recovery. However, clinical and microbiological assessments should complement PCT levels due to potential false results [64].

An ideal biomarker with high clinical accuracy for sepsis diagnosis is still needed. Novel biomarkers, such as *Pancreatic Stone Protein (PSP)* [65–67]*, Presepsin, and Mid-regional Pro-adrenomedullin (MR-proADM)* [68], showing early elevation in response to sepsis, are under study. Future clinical trials are necessary to further verify their utility in clinical practice.

### Sources of infection

Infections are more prevalent in older individuals correlating with increased hospitalization and mortality, particularly in those over 85 years [69]. Lower respiratory tract and urinary tract infections (UTIs) are predominant both in community and health care associated infections (HAI) [49, 70]. Among 308 elderly individuals, respiratory tract infections represented 49.7%, urinary tract infections (UTIs) 33.8%, blood stream infections (BSIs) 21.1%, and surgical site infections 4.9% [9].

Pneumonia, a severe respiratory tract infection, can be challenging to diagnose because of atypical symptoms and difficulty in obtaining accurate chest radiographs due to physical limitations. Lung ultrasound and CT scanner can aid in the diagnosis, while bronchoscopy and BAL are recommended for immunocompromised and more critical patients. Aspiration pneumonia, with a higher mortality rate, is prevalent among older adults especially if impaired swallowing, intubation or in general anaesthesia’s postoperative phase. About 76% of aspiration pneumonia-related deaths occur in patients aged 75 years or older [71]. Common microorganisms include Streptococcus pneumoniae, Staphylococcus aureus, Haemophilus influenzae, and Enterobacteriaceae. In case of poor dental health, anaerobic microorganisms should also be considered [72]. In HAI the pathogens involved are mainly gram-negative bacteria (many of which are MDR) [70]. COVID-19 was a major complication in the older population, leading to high mortality rates, particularly among those requiring invasive ventilation [73].

UTI diagnosis is challenging due to overlapping symptoms, the presence of asymptomatic bacteriuria (ASB) and difficulties in obtaining uncontaminated urine. Approximately 15–50% of patients aged 80 and older have ASB, and over 50% of antibiotic treatments for ASB are unnecessary [74–76]. In urinary sepsis, E. coli is the most common microorganism, but catheter-associated infections are polymicrobial, including *Proteus* spp., *Klebsiella* spp., *E. faecalis* and *Pseudomonas* spp. [77].

BSIs are common and fatal in older patients, with around half of all cases occurring in this age group. Case fatality rates peak at 50–60% for individuals over 85 years. Older people face increased risks for Gram-negative infections, urinary source infections, and antimicrobial resistance, frequently healthcare-associated [78–80]. MDR microorganisms, pose significant challenges and may lead to treatment failures. In a Spanish cohort with healthcare-associated bacteremic UTIs, over 61% had MDR microorganisms, and over 75% were elderly [81]. Removing unnecessary urinary catheters could reduce a significant portion of these BSIs.

Skin and soft tissue infections (SSTIs) are prevalent in older adults, exhibiting a wide clinical spectrum from mild infections to life-threatening diseases. Prognosis worsens with comorbidities, such as heart failure, diabetes mellitus, and malnutrition. SSTIs pose a notable challenge in treatment, especially in acute care hospitals and long-term care facilities, where their prevalence is significant (10.9% and 17%, respectively) [70, 82, 83]. Common bacteria associated with SSTIs in this demographic include Streptococcus spp., Staphylococcus spp., and Pseudomonas aeruginosa. Screening for risk factors associated with methicillin-resistant *Staphylococcus aureus* (MRSA) is crucial.

Hospital-acquired infections (HAIs) pose serious health risks to the older population, resulting in longer hospital stays, extended antibiotic therapy, significant mortality, and higher healthcare costs. HAIs are the primary cause of death in one-third of individuals aged 65 and over. MDR microorganisms make infection prevention and control measures crucial [70].

### Management of sepsis

Treatment of older individuals with sepsis/septic shock should adhere to the Surviving Sepsis Campaign (SSC) International Guidelines [84], but the following items require special attention.

#### Antibiotic therapy

Empirical antibiotic should consider common pathogens, their susceptibility to antimicrobials and resistance patterns. The risk of infections by MDR microorganisms is notable in this demographic due to frequent healthcare exposure [70]. In addition, older individuals face an elevated risk of fungal infections due to age-related changes, compromised immune status, catheter use, prolonged antibiotic use, and treatments, such as corticosteroids and chemotherapy [85, 86].

Selecting and dosing antibiotics is challenging due to factors, such as comorbidities, drug pharmacokinetics (PK) and pharmacodynamics (PD), polypharmacy and risk of drug interactions [87, 88]. Age-related changes in organ function, body composition, renal clearance, hepatic metabolism, and drug distribution significantly influence antibiotic PK and PD [49, 89, 90]. With aging, the decrease in body water percentage reduces the distribution volume for hydrophilic drugs (e.g., β-lactams, glycopeptides, aminoglycosides, azoles), leading to a faster increase in plasma concentrations. Conversely, a relative increase in adipose tissue raises the distribution volume for lipophilic drugs (e.g., macrolides, fluoroquinolones), prolonging their half-life and leading to lower tissue concentrations [90]. Age-related liver and renal declines affect drug half-life and elimination [87, 91–94]. Adjustments for antibiotics in reduced renal function involve considering bacterial killing type. For concentration-dependent antibiotics, increase dosing intervals to prevent overdosing; for time-dependent ones, reduce the dose while maintaining the interval.

Morphological and functional changes such as delayed gastric emptying, reduced splanchnic blood flow and altered gastric pH can affect the bioavailability of orally administered drugs [87, 89, 90, 92].

#### Resuscitation

In fluid resuscitation and hemodynamic support, careful fluid management is crucial, considering comorbidities and age-related changes in autoregulation. While guidelines propose a target mean arterial pressure (MAP) of ≥ 65 mm Hg, older patients with chronic hypertension may require higher MAP targets to prevent acute kidney injury [95, 96]. Dehydration is common in older adults, often necessitating an initial 500 mL crystalloid bolus. However, protocolized resuscitation, such as 30 mL/kg of intravenous crystalloid within 3 h, may be detrimental in patients with cardiac impairment or chronic kidney disease [5]. Excessive fluid therapy can lead to impaired outcomes, emphasizing the need for a dynamic evaluation of fluid response. Customized assessment of perfusion indicators, including mental status, diuresis, circulatory assessment, pulse rate, blood pressure, capillary refill, and point-of-care echocardiography, is crucial for monitoring and treatment decisions. Initiating de-resuscitation promptly with diuretics is essential.

The ideal hemoglobin transfusion threshold in older septic patients is undefined and may differ from that in young adults. Anemia is increasingly prevalent in the aging population, affecting over 10% of those aged 65 and older, with nearly two-thirds of critically ill patients in ICUs experiencing anemia. In sepsis, anemia's multifactorial causes include reduced red blood cell production, stress-induced bleeding, hemodilution, recurrent blood withdrawal, impaired iron metabolism and hemolysis. A study of 815 older septic patients revealed over 20% had hemoglobin levels below 10 g/dL on admission, doubling during the first week. Although initial hemoglobin strongly correlated with in-hospital mortality, blood transfusions, administered to 8.3% of patients, were not an independent predictor of mortality [97] A recent meta-analysis focusing on older adults suggests higher hemoglobin thresholds result in lower mortality and fewer cardiac complications, considering age-related declines in cardiac output affecting oxygen delivery [98]. Ongoing debates and trials explore anemia management, transfusion thresholds, and frequency.

#### Additional factors

Individualized sedation protocols, short-acting drugs, and nonpharmacologic approaches for managing pain, agitation and delirium significantly enhance outcomes in critically ill adults [39]. The PADIS guidelines, crucial for all patients, are especially important for older individuals. They advocate for shorter mechanical ventilation (MV), early mobilization, and notably contribute to reducing sarcopenia and delirium incidence in older patients. Delirium rates can reach 80% in ventilated older patients, compared to 33% in general medical units, significantly increasing the risk of persistent cognitive impairment post-discharge. Up to 70% may experience prolonged cognitive impairment within a year post-hospitalization, with around 10% developing dementia [99, 100]. Ventilated patients face a 30% higher likelihood of needing assistance with activities of daily living (ADLs) compared to non-ventilated individuals.

Non-invasive ventilation (NIV) reduces risks associated with mechanical ventilation and eases discomfort in critically ill older patients. While guidelines primarily recommend NIV for acute COPD exacerbation with hypercapnia and acute respiratory failure due to pulmonary oedema, it is not the preferred initial therapy for hypoxemic respiratory failure from pneumonia, because the potential need for intubation post-NIV failure carries severe clinical implications and a high risk of death. An analysis [101] compared NIV as the primary mode of respiratory support in two large observational studies with 1986 patients aged ≥ 80 (1292 from the VIP2 study, pre-pandemic era and 694 from the COVIP study, during pandemic). Those with COVID-19 ARDS treated primarily with NIV were less likely to survive 30 days after ICU admission, despite being less frail. This discrepancy may be linked to the study population, as VIP2 included patients with respiratory failure from COPD or pulmonary oedema. In addition, the risk of NIV failure quadrupled during the COVID-19 pandemic.

Steroid use in sepsis is a subject of debate due to conflicting evidence regarding its impact on mortality [102–104]. The SSC guidelines recommend intravenous hydrocortisone at a dose of 200 mg per day if adequate fluid resuscitation and vasopressor therapy fail to restore hemodynamic stability or if adrenal impairment is suspected. Gradual reduction is advised when vasopressor support is no longer needed. The decision to use steroids in septic older patients should be individualized, considering the patient's overall health, comorbidities, and the specific circumstances of their sepsis.

Finally, Impaired glucose control, thrombotic events and stress ulcers are more frequent in the older population. Therefore, glucose control should be monitored, and insulin therapy should be initiated promptly if hyperglycaemia is detected, although optimal target levels are not well-defined. Pharmacologic thromboembolic prophylaxis with LMWH, considering renal function and bleeding risks, as well as stress ulcer prophylaxis [105], is recommended for older patients with sepsis.

In addition to specific sepsis treatments, incorporating multidisciplinary interventions is crucial [106–108]. Utilizing a comprehensive geriatric assessment to understand an older patient’s medical, psychosocial, and functional capabilities can enhance their functional status, prevent institutionalization, and reduce mortality for those admitted to the hospital. High-quality geriatric nursing, including falls prevention, nutrition, and physiotherapy, remains important beyond the acute illness phase.

### Outcomes

Sepsis has a profound impact on the senior population, leading to significant morbidity and mortality [41, 77, 109]. The financial strain on healthcare systems is significant, with extensive healthcare resource utilization both before and after ICU admission [110–115].

In patients aged ≥ 65, in-hospital mortality ranges from 30 to 60%, escalating to 40–80% in those aged 80 and above [49]. A systematic review of very old septic patients in the ICU reports mortality rates of 43% in the ICU, 47% in the hospital, and 68% one year after ICU admission [116]

An analysis of the Intensive Care Over Nations (ICON) database, focusing on patients above 50 years, reveals age-related differences in sepsis outcomes. Hospital mortality increases with age, doubling in patients over 80 compared to those under 50 years (49.3% vs. 25.2%, *p* < 0.05). Mortality sees a maximum rate increase of about 0.75% per year between the ages of 71 and 77 years. Multilevel analysis confirms age > 70 years as an independent risk factor for mortality [117].

Despite age often being considered an independent risk factor for mortality and morbidity [118–120], emerging research underscores the crucial roles of other factors, such as frailty, disease severity, and comorbid conditions [26, 121–125]. Post-hoc analyses of the VIP-1 and VIP-2 studies, examining patients aged 80 and over admitted to the ICU with sepsis, show ICU mortality rates of 31% and 41%, with 30-day and 6-month mortality rates of 45% and 54%, respectively [121, 122] (Table 1). Sepsis as admission diagnosis did not maintain an independent link to mortality after adjusting for organ dysfunction. Frailty, advanced age, and SOFA score emerged as key independent prognostic factors for adverse outcomes (Table 2).

**Table 1:** Characteristics of older patients (≥ 80 years) admitted to the ICU with sepsis diagnosis in VIP-1 and VIP-2 studies

Cohorts	Sepsis VIP-1	Sepsis VIP-2
*N* (%)	493/3869 (12.7%)	532/3596 (14.8%)
Age (years)	83 (81–86)	84 (81–86)
Gender (male)	265 (53.8%)	298 (56%)
SOFA score at admission	9 (6–12)	9 (6–11)
ICU LOS (days)	3.54 (1.5–8)	4.77 (2–9)
Frailty (CFS)		
Fit (CFS 1–3)	165 (33.5%)	195 (36.7%)
Vulnerable (CFS 4)	76 (15.4%)	89 (16.7%)
Frail (5–9)	252 (51.1%)	248 (46.6%)
ICU interventions		
Mechanical ventilation	234 (47.5%)	260 (49%)
Non-invasive ventilation	108 (21.9%)	86 (16.2%)
Vasoactive drugs	405 (82.2%)	456 (85.9%)
Renal replacement techniques	86 (17.4%)	109 (20.6%)
Limitations of care		
Withholding	108 (21.9%)	186 (35.6%)
Withdrawing	76 (15.4%)	79 (15.1%)
Mortality		
ICU	154 (31.2%)	166 (41.4%)
30 days	220 (44.6%)	
6 months		286 (54%)

**Table 2:** Multivariate analysis (Cox). Predictors of 30-day mortality (VIP-1 study) and 6-month mortality (VIP-2 study) in older patients (≥ 80 years), admitted to the ICU with sepsis

	30-day mortality HR (95% CI)	*P* value	6-month mortality HR (95% CI)	*P* value
Age (per 5-year increase)	1.16 (1.09–1.25)	< 0.0001	1.16 (1.09–1.25)	< 0.0001
Frailty (CFS > 4)	1.47 (1.31–1.66)	< 0.0001	1.34 (1.18–1.51)	< 0.0001
SOFA score (per one-point increase)	1.13 (1.12–1.14)	< 0.0001	1.16 (1.14–1.17)	< 0.0001
Sepsis	0.99 (0.86–1.15)	0.92	0.89 (0.77–1.02)	0.09

Advancements in sepsis management have led to a reduction in sepsis-associated mortality. [2, 10, 126–131], even among the older population [132]. However, older sepsis survivors face worse long-term outcomes, including greater cognitive and functional decline, an increase risk of hospital readmission, and a higher likelihood of discharge to long-term care facilities [18, 115, 133–135].

Post-intensive care syndrome (PICS) symptoms, prevalent among older sepsis survivors, include muscle weakness, fatigue, cognitive decline, sleep disturbances, emotional distress, and swallowing problems [125, 136, 137]. Another term, possibly more specific, is post-sepsis syndrome (PSS) [138]. Factors such as pre-existing co-morbidity and frailty, polypharmacy, delirium during hospitalization and injury induced by sepsis [134, 138] can exacerbate outcomes.

Ongoing efforts to improve sepsis management, including early recognition, prompt source control, and timely antibiotic administration are crucial. In addition, adopting a multi-faceted approach to improve long-term outcomes for survivors is essential.

### Goals of care

Predicting survival or future quality of life for older individuals poses challenges due to the substantial biological and functional heterogeneity in this demographic. Ethical and legal frameworks vary globally, influencing diverse management approaches among healthcare professionals shaped by geographical locations and cultures. In the absence of robust evidence guiding patient management, decisions regarding the proportionality of intensive care often stem from personal preferences and experience [139].

Key criteria for ICU admission include the condition's reversibility, emphasizing both survival and maintaining a similar quality of life. Medical treatment should align with the patient's wishes and prioritize their well-being, considering the burden vs. the chance of recovery. Recognizing the patient’s perspective on aging, health, and disease is crucial, as some prioritize quality of dying over life-prolonging measures [122, 127, 132]. In uncertain cases, a therapeutic trial is recommended, with its duration remaining undefined and contingent on the patient’s response. If irreversibility becomes clear, discussions with the patient, surrogates, and colleagues guide decisions on excluding treatments causing suffering. Divergent opinions require additional time for clarity.

**Fig. 5:**
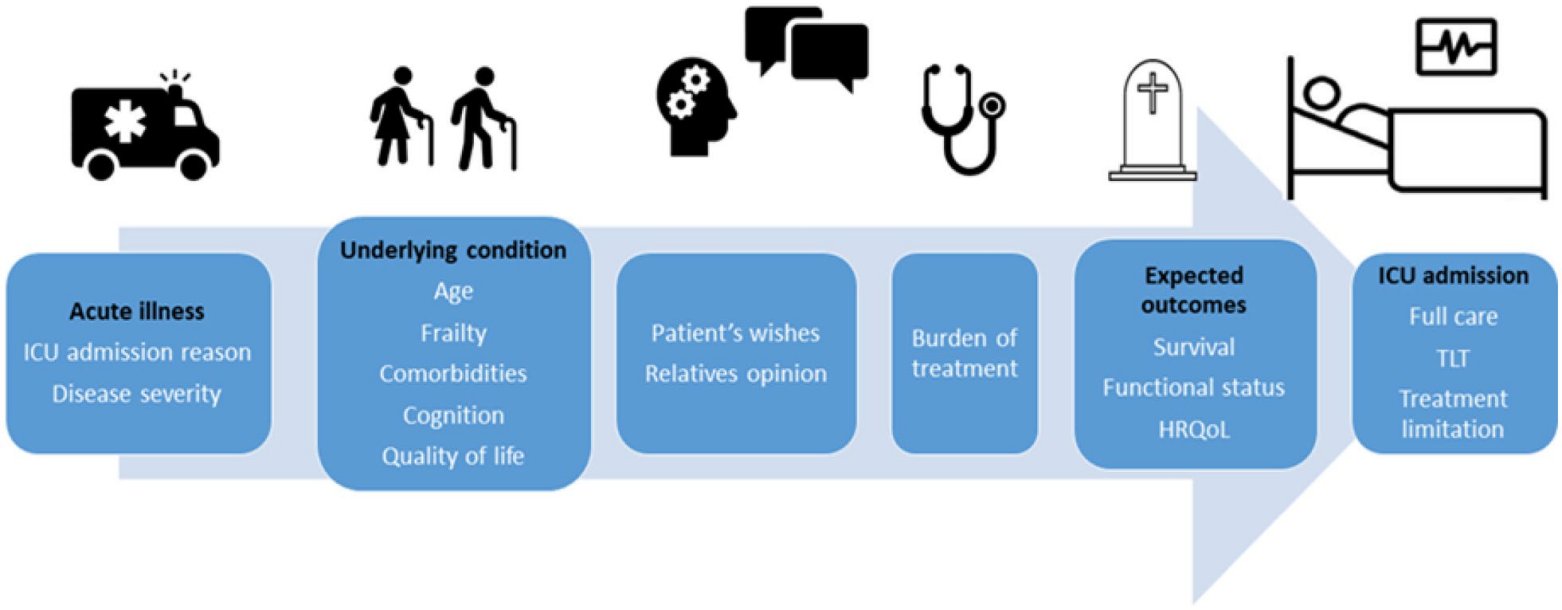
Triage considerations for the older septic patient. *HRQoL* Health-Related Quality of Life, *TLT* Time Limited (ICU) Trial

Decisions to limit life-sustaining treatment (LST) should account for baseline status, quality of life, survival potential, functional outcomes, and treatment burden. Mousai et al. [140] illustrate that integrating clinical phenotypes with cultural factors and information about critical care course enhances predictive discrimination accuracy for LST in very old ICU patients. Clinicians can make these decisions either before ICU admission or as the patient’s condition evolves. Family involvement and regular discussions about the patient’s condition are essential. A framework encompassing physical and cognitive status, quality of life, survival likelihood, functional performance, preferences, and treatment burden guides decisions for intensive care in older patients (Fig. 5) [18, 84, 134].

## Conclusions

Sepsis poses a significant threat to the senior population. However, current research on this demographic remains insufficient. It is imperative to raise awareness, educate healthcare professionals, implement preventive measures, and deliver timely and appropriate care to improve outcomes.

The insights from the VIP-1 and VIP-2 studies prompt a reassessment of sepsis as a standalone contributor to mortality, emphasizing the importance of understanding and addressing comorbid geriatric conditions to enhance patient resilience and overall prognosis. In addition, it is crucial to inquire about the patient's preferences and establish a personalized treatment plan that considers their potential for recovery with an acceptable HRQoL and functional outcomes.

The aim ahead is to recognize the gaps and limitations in current research while determining short- and long-term priorities. These priorities should extend beyond merely reducing sepsis mortality to gaining insights into, and enhancing, the HRQoL of sepsis survivors.

## Data Availability

Not applicable.

## Abbreviations

ASB: Asymptomatic bacteriuria
COPD: Chronic obstructive pulmonary disease
CFS: Clinical Frailty Scale
HRQoL: Health-related quality of life
ICU: Intensive care unit
IMV: Invasive mechanical ventilation
LOS: Length of stay
LST: Life sustaining treatment
MDR: Multi-drug resistant
NIV: Non-invasive ventilation
PCT: Procalcitonin
PICS: Post-intensive care syndrome
RRT: Renal replacement therapy
SOFA: Sequential Organ Failure Assessment
TLT: Time Limited (ICU) Trial
UTI: Urinary tract infection
VIPs: Very Old Intensive Care Patients
